# The Relationship Between In-Hospital Mortality and Frontal QRS-T Angle in Patients With COVID-19

**DOI:** 10.7759/cureus.28506

**Published:** 2022-08-28

**Authors:** Ercan Tastan, Ümit İnci

**Affiliations:** 1 Cardiology, Diyarbakir Gazi Yasargil Education and Research Hospital, Diyarbakır, TUR; 2 Cardiology, Gazi Yaşargil Training and Research Hospital, Diyarbakır, TUR

**Keywords:** echo cardiogram, ekg abnormalities, in-hospital mortality, frontal qrs-t angle, covid-19

## Abstract

Background

Recent studies have demonstrated that the frontal QRS-T angle, defined as the angle between the mean QRS and T vectors, is a strong independent predictor of mortality in patients with cardiovascular disease and in the normal population. In this study, we aimed to investigate the relationship between frontal QRS-T angle and in-hospital mortality in COVID-19 patients.

Methods

A total of consecutive 532 patients with positive polymerase chain reaction (PCR) tests were enrolled. The patients were divided into two groups as in-hospital mortality and survival groups. Frontal QRS-T angle was automatically calculated from the admission electrocardiography (ECG).

Results

The median age in the study population was 62 (49-72) years and 273 (51.4%) of the patients were male. The median frontal QRS-T angle was 40 degrees (20-67 IQR) in the in-hospital mortality group, while it was 27 (11-48 IQR) in the survival group (p=0.001). In multivariable logistic regression analysis, frontal QRS-T angle was found to be an independent predictor of mortality (Odds ratio (OR):1.01, 95% Confidence interval (CI):1.00-1.02, p=0.036).

Conclusion

Frontal QRS-T angle, which was observed wider in the in-hospital mortality group, was found to be associated with in-hospital mortality in patients hospitalized for COVID-19.

## Introduction

COVID-19 disease caused by SARS-CoV-2 (Severe acute respiratory syndrome coronavirus 2) remains a significant cause of morbidity and mortality. While COVID-19 primarily affects the lungs, causing pneumonia and severe acute respiratory distress syndrome (ARDS), it also affects multiple organs and systems particularly the cardiovascular system. Cardiovascular complications such as dysrhythmia, acute coronary syndrome, myocarditis, and heart failure can be seen in COVID-19 infection [1].

The frontal QRS-T angle, between ventricular depolarization and repolarization axles, which can be easily measured in 12-lead electrocardiography (ECG), is a marker of ventricular repolarization heterogeneity [2]. Increasing the frontal QRS-T angle has been found to be a predictor of cardiac and all-cause mortality in many studies conducted in patients with heart failure, acute coronary syndrome, myocarditis, and in the normal population [2,3-11]. There is insufficient data about the relationship between frontal QRS-T angle and mortality in COVID-19 patients. In this study, we aimed to investigate the relationship between frontal QRS-T angle and in-hospital mortality in COVID-19 patients.

## Materials and methods

In this single-center retrospective study, patients hospitalized in Diyarbakır Gazi Yaşargil Training and Research Hospital due to COVID-19 infection between 1-31 August 2020 were screened. Those with admission ECG and positive real-time polymerase chain reaction (PCR) for COVID-19 were included in the study. Patients with negative PCR, atrial fibrillation (due to differences in size in ECG), bundle branch block, pacemaker rhythm and patients whose ECG could not be evaluated were excluded from the study. A total of 930 patients were screened for the study, and after exclusion criteria, the remaining 532 patients were included in the study. Patients were divided into two groups as in-hospital mortality and survival groups.

The demographic, clinical characteristics, laboratory parameters, comorbidities, and hospitalization time of the patients during hospitalization were obtained from the electronic medical records of the hospital and national electronic medical record system.

The study was approved by the local institutional ethics committee. The study protocol conformed to the Declaration of Helsinki.

Electrocardiographic assessment

12-lead ECG was taken with 25mm/sec speed and 10mm/mv calibration when patients were admitted to the hospital. The frontal QRS-T angle was defined as the absolute value of the difference between the frontal plane QRS and T axis. It was set to the minimum angle if the angle was >180 degrees (360 degrees-angle). Frontal QRS-T angle was obtained from automated ECG device data. QT intervals were corrected for the effects of heart rate using a modified Bazett formula (QTc=QT/(R-R)1/2 ).

Statistics

The continuous variables were presented as a median interquartile range (IQR) (25-75%) owing to their non-normal distribution. The histogram and Shapiro-Wilk test were used to verify the normal distribution of data. The categorical variables were expressed as percentages. Chi-square test was used to compare categorical variables between groups. Continuous variables were compared by Mann-Whitney U tests. Univariable and multivariate logistic regression analyzes were performed to determine the effect of frontal QRS-T angle on in-hospital mortality. Variables with p-value < 0.1 in univariable analysis were added to the model in multivariable analysis. We selected predictive candidates based on previous studies and known or plausible associations with exposure to COVID-19 infection [12]. Values of p < 0.05 were considered to be statistically significant. The analysis of the data was carried out using SPSS (Statistical Package for Social Science for Windows)-24 packaged software (IBM Corp., Armonk, NY).

## Results

Five hundred thirty-two patients who had positive PCR tests were included in our study. The median age in the study population was 62 (49-72) years and 273 (51.4%) of the patients were male. In the study population, 38.4% had hypertension, 26.3% had diabetes mellitus, 15% had a history of coronary artery disease, and 6% had myocardial injury. Ninety-eight patients died during hospitalization (18.4%). Patients were divided into two groups as in-hospital mortality and survival groups. While frontal QRS-T angle was median 40 (20-67 IQR) degrees in the in-hospital mortality group, the median was 27 (11-48 IQR) degrees in the survival group and the difference between the two groups was statistically significant (p<0.001). It was found that ventricular tachycardia developed in two patients in the mortality group. The demographic, clinical characteristics, laboratory and electrocardiographic findings of the study population were shown in Table 1.

**Table 1:** The demographic, clinical characteristics, laboratory and electrocardiographic findings of the study population Continuous variables are presented given as median (interquartile range) and categorical variables were expressed as number (%). ACE/ARB: Angiotensin-converting enzyme inhibitors/angiotensin receptor blockers

	Total (n=532)	In-hospital mortality (n=98)	Survival (n=434)	p-value
Age (years)	62 (49-72)	70 (62-78)	59 (46-70)	<0.001
Gender (men)	273 (51.3%)	59 (60.0%)	214 (49.3%)	0.051
Hypertension, n (%)	204 (38.4%)	52 (53.0%)	152 (35.0%)	0.001
Ischemic heart disease, n (%)	80 (15.0%)	21 (21.4%)	59 (13.6%)	0.071
Heart failure, n (%)	20 (3.7%)	8 (8.1%)	12 (2.7%)	0.018
Chronic kidney failure, n (%)	19 (3.6%)	7 (7.1%)	12 (2.8%)	0.062
Cerebrovascular disease, n (%)	18 (3.4%)	4 (4.0%)	14 (3.2%)	0.756
Diabetes mellitus, n (%)	140 (26.3%)	32 (32.6%)	108 (24.9%)	0.115
Chronic obstructive pulmonary disease, n (%)	24 (4.5%)	8 (8.1%)	16 (3.6%)	0.062
Myocardial injury, n (%)	33 (6%)	18 (18.3%)	15 (3.4%)	<0.001
Creatinine, mg/dl	0.91 (0.76-1.20)	1.16 (0.80-1.71)	0.89 (0.75-1.12)	<0.001
Potassium, meq/L	4.13 (3.80-4.49)	4.25 (3.90-4.77)	4.11 (3.80-4.42)	0.005
Calcium, mg/dl	8.2 (7.8-8.6)	7.9 (7.5-8.3)	8.3 (7.8-8.7)	<0.001
Albumin, g/L	33 (29-36)	29.5 (27-32)	33 (30-37)	<0.001
Aspartate Aminotransferase, IU/L	32 (24-47)	38 (28-54)	30 (23-45)	<0.001
Alanine Aminotransferase, IU/L	23 (17-39)	24 (18-39)	23 (17-39)	0.563
Lactate dehydrogenase, U/L	328 (261-430)	423 (315-592)	317 (256-397)	<0.001
Ferritin, ng/ml	439 (222-857)	650 (277-1190)	410 (202-758)	<0.001
C-reactive protein, mg/dL	80 (36-129)	136 (80-200)	68 (32-116)	<0.001
Procalcitonin, ng/ml	0.1 (0.05-0.25)	0.36 (0.13-1.14)	0.08 (0.04-0.16)	<0.001
D-dimer, ng/mL	274 (169-439)	493 (270-1186)	249 (163-366)	<0.001
Troponin, ng/mL	0.1 (0.1-0.1)	0.1 (0.1-0.1)	0.1 (0.1-0.1)	<0.001
White blood cell, 10^3^/uL	6.9 (5.2-9.5)	9.3 (6.2-13.4)	6.6 (5.1-8.8)	<0.001
Neutrophil, 10^3^/uL	5.2 (3.6-7.8)	7.9 (5.1-11.4)	4.8 (3.4-7)	<0.001
Lymphocyte, 10^3^/uL	1.1 (0.8-1.4)	0.76 (0.6-1.1)	1.2 (0.86-1.48)	<0.001
Hemoglobin, gr/dL	13.4 (12.2-14.4)	12.9 (11.7-14.2)	13.4 (12.4-14.5)	0.008
Platelet, 10^3^/uL	206 (161-259)	195 (159-262)	208 (161-258)	0.639
Oxygen saturation, %	90 (84-93)	75 (70-82)	91 (87-94)	<0.001
Systolic blood pressure, mmHg	120 (110-130)	120 (104-130)	120 (110-125)	0.104
Diastolic blood pressure, mmHg	70 (65-80)	70 (60-80)	70 (70-80)	0.519
Hospitalization time, (day)	7 (6-11)	10 (6-12)	7 (6-10)	<0.001
Admission heart rate (beats/min)	91 (81-100)	95 (86-107)	90 (80-100)	0.001
QRS duration (ms)	85 (80-96)	80 (80-90)	86 (80-96)	0.045
QT interval (ms)	360 (330-380)	340 (320-380)	360 (340-380)	0.078
QTc interval (ms)	433 (413-454)	434 (412-461)	433 (413-453)	0.578
Frontal QRS-T angle (degree)	30 (12-52)	40 (20-67)	27 (11-48)	0.001
ACE/ARB, n (%)	166 (31.2%)	39 (39.8%)	127 (29.3%)	0.042
B blocker, n (%)	82 (15.4%)	24 (24.5%)	58 (13.4%)	0.009
Calcium channel blocker, n (%)	96 (18%)	30 (31%)	66 (15%)	0.001
Thiazide diuretic, n (%)	99 (18.6%)	21 (21.5%)	78 (18%)	0.515
Loop diuretic, n (%)	13 (2.4%)	9 (9.1%)	14 (3.2%)	0.023

In multivariable logistic regression analysis, neutrophil-lymphocyte ratio (OR: 1.08, 95% CI: 1.03-1.13, p=0.002), age (OR: 1.06, 95% CI: 1.03-1.09, p<0.001), creatinine (OR: 1.30, 95% CI: 1.10-1.54, p=0.001), D-dimer (OR: 1.03, 95% CI: 1.01-1.05, p=0.015), oxygen saturation (OR: 0.79, 95% CI: 0.75-0.84, p<0.001) were found to be independent predictors of in-hospital mortality. In addition, frontal QRS-T angle was found to be an another independent predictor of mortality (Odds ratio (OR): 1.01, 95% Confidence interval (CI): 1.00-1.02, p=0.036) (Table 2).

**Table 2:** Univariable and multivariable logistic regression analysis to identify independent predictors of in-hospital mortality CI: Confidence interval, COPD: Chronic obstructive pulmonary disease, NLR: Neutrophil/Lymphocyte ratio, LDH: Lactate dehydrogenase

	Univariable analysis			Multivariable analysis		
	Odds ratio	95% CI	p-value	Odds ratio	95% CI	p-value
Age (years)	1.07	1.05-1.09	<0.001	1.06	1.03-1.09	<0.001
Gender (female)	0.64	0.41-1.01	0.052	0.88	0.42-1.85	0.740
Coronary artery disease	1.73	0.99-3.02	0.052	0.97	0.36-2.64	0.951
Hypertension	2.19	1.40-3.40	<0.001	0.75	0.34-1.65	0.480
Diabetes mellitus	1.53	0.96-2.46	0.076	1.74	0.80-3.77	0.160
Heart failure	3.13	1.24-7.87	0.016	1.73	0.35-8.57	0.500
COPD	2.32	0.96-5.59	0.060	1.72	0.35-8.46	0.503
Myocardial injury	6.27	3.03-12.95	<0.001	2.31	0.63-8.48	0.204
NLR	1.15	1.10-1.19	<0.001	1.08	1.03-1.13	0.002
D-dimer (for every 100 unit increments)	1.04	1.02-1.07	<0.001	1.03	1.01-1.05	0.015
LDH (for every 10 unit increments)	1.04	1.02-1.05	<0.001	1.01	0.99-1.03	0.537
Creatinine (mg/dL)	1.33	1.14-1.56	<0.001	1.30	1.10-1.54	<0.002
Oxygen saturation, (%)	0.79	0.75-0.82	<0.001	0.79	0.75-0.84	<0.001
Frontal QRS-T angle (degree)	1.01	1.01-1.02	<0.001	1.01	1.00-1.02	0.036
Cerebrovascular disease	1.28	0.41-3.97	0.673			

## Discussion

Our study is one of the few studies investigating the relationship between frontal QRS-T angle and mortality in COVID-19 patients. The results of this study showed that the frontal QRS-T angle was wider in the mortality group than the survival group and the frontal QRS-T angle was an independent predictor of in-hospital mortality in patients hospitalized for COVID-19.

SARS-CoV-2 mainly affects the respiratory system and can occur in a wide range from asymptomatic subclinical infection to ARDS. SARS-COV-2, which causes complications in many organs and tissues as well as the respiratory system, can also cause concomitant cardiovascular complications. Some patients with COVID-19 may have symptomatic heart disease such as myocarditis, cardiac arrhythmias including sudden cardiac arrest, myocardial injury, acute coronary syndrome, and heart failure. Some patients may have abnormalities in laboratory and imaging tests (such as serum cardiac troponin elevation, asymptomatic cardiac arrhythmias, or abnormalities on cardiac imaging) without symptoms of heart disease, and some patients do not have any signs of heart disease [1].

The QRS-T angle, defined as the angle between the QRS and T axis, shows the main direction of the electrical activity of the heart during ventricular depolarization and repolarization. The wide QRS-T angle is considered an independent and strong predictor for cardiac morbidity and mortality compared to conventional electrocardiographic predictors of cardiovascular risk (such as long QT interval, left ventricular hypertrophy, and ST segment depression) [13]. Frontal QRS-T angle was found to be associated with all-cause death, cardiac death, and arrhythmias in patients with ischemic heart failure, non-ischemic heart failure, heart failure with preserved ejection fraction, myocarditis, acute myocardial infarction, and in the normal population [2,3-11]. In addition, patients with the coronary slow-flow [14] and severe coronary artery disease [15] were found to have wider frontal QRS-T angles compared to those without. In our study, the frontal QRS-T angle was observed to be wider in the mortality group. And in the regression analysis, it was found that the risk of mortality was significantly related to the widening of the frontal QRS-T angle.

Although the pathophysiology of cardiovascular system involvement in COVID-19 is not fully known, many mechanisms have been suggested. It is known that viral infections are associated with metabolic disorders, myocardial inflammation, sympathetic nervous system activation, and cause arrhythmias [1]. One of the important causes of myocardial injury in COVID-19 disease is myocarditis, and the cause of myocardial inflammation is not known exactly [1]. Since COVID-19 disease is a systemic infection, it may lead to erosion or rupture of pre-existing plaques. As a result, type-1 myocardial infarction may develop [16]. Pneumonia/ARDS-associated hypoxia can trigger oxidative stress in endothelial cells or myocardiocytes in several ways and cause supply-demand imbalance between myocardium and coronary vessels [17]. In addition, it has been suggested that coronary microvascular dysfunction develops with many mechanisms in COVID-19 patients [18]. The decrease in angiotensin-converting enzyme-2 (ang 2) level in small coronary vessels in COVID-19 disease causes an increase in ang 2/ang (1-7) level. And this situation causes renin-angiotensin-aldosterone system imbalance. As the Ang 2/ang(1-7) ratio increases in small coronary vessels, the ang 2-AT1R pathway becomes active. As a result, vasoconstriction, increased vasopressin release, and autonomic nerve dysfunction may develop [16]. In addition, systemic inflammation and hypercoagulability in COVID-19 patients may cause a slowdown in coronary blood flow [18]. Moreover, cardiac involvement has been shown in COVID-19 patients without cardiac symptoms. In a study by Huang et al., it was shown that seven of 26 patients developed focal and diffuse fibrosis in cardiac magnetic resonance imaging (MRI) performed in the later periods, although there were no symptoms during COVID-19 disease [19]. In addition, it has been reported that a patient without a history of myocarditis during COVID-19 disease developed diffuse myocardial fibrosis in cardiac MRI taken three months later with complaints of atypical chest pain and palpitations [20]. Although the reason for the widening of the frontal QRS-T angle in COVID-19 patients is not known exactly, heterogeneous areas in myocardial tissue may develop with the cardiovascular complications and mechanisms mentioned above. Myocardial repolarization and depolarization abnormalities caused by this heterogeneity may cause a widening of the frontal QRS-T angle.

Frontal QRS-T angle, which was previously shown to be of prognostic importance in the non-COVID-19 population and can be easily calculated by 12-lead ECG, was also associated with in-hospital mortality in COVID-19 patients. In this context, calculating the frontal QRS-T angle in patients hospitalized with COVID-19 may contribute to the identification of critical patients and their follow-up. There is a need for multicenter and prospective studies on the frontal QRS-T angle in COVID-19 patients.

The most important limitation of our study is its single-center and retrospective design. Another limitation is that obesity could not be included in the regression analysis due to the lack of body mass index data.

## Conclusions

Frontal QRS-T angle, which was observed wider in the in-hospital mortality group, was found to be associated with in-hospital mortality in patients hospitalized for COVID-19. The frontal QRS-T angle, which can be easily obtained from surface ECG, can be used for predicting prognosis in these patients.
